# A Color Gamut Description Algorithm for Liquid Crystal Displays in *CIELAB* Space

**DOI:** 10.1155/2014/671964

**Published:** 2014-04-24

**Authors:** Bangyong Sun, Han Liu, Wenli Li, Shisheng Zhou

**Affiliations:** ^1^School of Printing and Packing, Xi'an University of Technology, Xi'an 710048, China; ^2^School of Automation and Information Engineering, Xi'an University of Technology, Xi'an 710048, China

## Abstract

Because the accuracy of gamut boundary description is significant for gamut mapping process, a gamut boundary calculating method for *LCD* monitors is proposed in this paper. Within most of the previous gamut boundary calculation algorithms, the gamut boundary is calculated in *CIELAB* space directly, and part of inside-gamut points are mistaken for the boundary points. While, in the new proposed algorithm, the points on the surface of *RGB* cube are selected as the boundary points, and then converted and described in *CIELAB* color space. Thus, in our algorithm, the true gamut boundary points are found and a more accurate gamut boundary is described. In experiment, a Toshiba *LCD* monitor's 3D *CIELAB* gamut for evaluation is firstly described which has regular-shaped outer surface, and then two 2D gamut boundaries (* CIE*-a*b* boundary and * CIE*-C*L* boundary) are calculated which are often used in gamut mapping process. When our algorithm is compared with several famous gamut calculating algorithms, the gamut volumes are very close, which indicates that our algorithm's accuracy is precise and acceptable.

## 1. Introduction

Different color devices generally have different color gamuts because of their specific coloring parameters, such as the coloring principle (additive and subtractive), colorant (pigment or dyers), substrates, and light source (D50, D65, or others). Thus, a color image often looks different when it is output by different color devices. For example, a brightly displayed photo on a monitor commonly loses some color information when printed on the paper. For the purpose of matching color image's visual effect between different devices, gamut mapping is often performed in digital imaging processing systems. Gamut mapping can be defined as the color rendering process which rearranges the colors from source device gamut to destination device gamut [1]. Because source and destination gamuts mismatch each other in some regions, there exist some colors which are inside the source gamut but outside of the destination gamut, and they must be clipped or compressed into the destination gamut.

Now there are dozens of gamut mapping algorithms (or GMAs) developed by different agencies[1]. For example, the* LCLIP* algorithm clips out-of-gamut colors onto the gamut boundary and leaves the chroma of in-gamut colors unchanged. The* CUSP* algorithm compresses colors towards a focus point, where the lightness and chroma are changed simultaneously. While for some universal GMAs, such as* CARISMA* or* GCUSP*, the mapping process is very complex but accurate. Because there are too many GMAs developed for the various color images, four specific GMAs are assigned within ICC workflow for convenience, which are absolute colorimetric, relative colorimetric, perceptual, and saturation [2].

Actually, these GMAs are sufficient to cope with all types of color images. However, the color inconsistency still occurs frequently when color images are transmitted from monitors to printers. As a matter of fact, one major reason which results in the gamut mapping errors comes from the gamut boundary calculation process, so it is significant to obtain the accurate gamut boundary before gamut mapping.

Gamut mapping is commonly performed in 2D coordinate, such as CIE-C*L* or CIE-a*b* coordinates [3]. The 2D gamut boundary is a cross-section from 3D* CIELAB* gamut, the errors within both the 3D gamut calculation process and the 2D gamut boundary calculation process will reduce the gamut mapping precision greatly.

Because there are some problems within the present gamut calculation algorithms (analyzed in Section 2), a 2D gamut boundary calculating algorithm is developed for gamut mapping process in this paper. In experiment an* LCD* monitor's gamut boundary is calculated and compared with other algorithms, and the experiment result shows that our algorithm is precise enough. In addition, it should be noted that the proposed algorithm is not only suitable for* RGB* monitors, but also for* CMY *(*CMYK*) printers or other color devices.

## 2. Different Types of Gamuts Employed in Gamut Mapping Process

In color reproduction systems, color gamut refers to the subset of colors which can be accurately represented in a given circumstance, such as within a given color image or by a certain color device. While the gamut boundary means the outer surface of the 3D gamut, or the outer contour line of the 2D gamut. Color gamut is often described in* CIELAB* space; this is mainly because* CIELAB* color spaces are independent of devices, and it is more perceptually uniform than* CIEXYZ* space.

Generally, there are three types of color gamuts described in* CIELAB* space, such as 3D* CIELAB* gamut, 2D CIE-a*b* gamut, and 2D CIE-C*L* gamut [4]. The first gamut is often used for viewing the overall gamut of color devices or images but rarely used in gamut mapping process directly. The two other gamuts are described in 2D coordinates which are often applied in gamut mapping process, while the main task of this paper is to calculate the 2D gamut's boundary for gamut mapping algorithms.

The CIE-a*b* gamut is a cross-section of 3D gamut along the constant lightness plane, and its boundary is the 3D gamut's outer intersection line with the same lightness. The CIE-C*L* gamut is a cross-section of 3D gamut along the constant hue-angle plane, and the boundary is the intersection line at the specified hue angle. Because hue information is generally more important than lightness, the third gamut boundary is most widely used during gamut mapping.

It is significant to obtain accurate description of the 3D gamut, because the 2D gamuts are calculated from it. Several analytical models are used to predict the 3D gamut with relatively few sample colors, such as the* Kubelka-Munk* function [5], and* Neugebauer* equations [6]. Moreover, Herzog considered the device's 3D gamut as a distorted hexahedron [7] and used a deformation degree function to simulate the actual device gamut. Huang and Zhao used* Zernike* polynomials to determine the boundary [8], and Wang and Xu used a two-step workflow to describe CRT monitor gamut boundaries [9]. On the whole, all these prediction algorithms are based on a specific analytical model and can get accurate gamut with few sample data. However, these methods cannot be applied to all types of color devices, because the analytical expression is highly restricted by the device status such as coloring principle, substrate, inks, and ambient light sources.

If a sufficient number of sample colors are supplied, the 3D gamut can be described with the empirical algorithms. These algorithms do not focus on analyzing the devices' coloring principle or the relationship between device color space and the corresponding* CIELAB* values; they straightly simulate the 3D gamut with the measured* CIELAB* values. Thus these methods can be used for all types of color devices, even for color images gamut calculation. Within these empirical algorithms, Balasubramanian and Dalal used a modified convex hull method to compute gamut, in a way of adjusting the concave gamut surfaces into convex [10]. Cholewo and Love used alpha-shape to describe the gamut surface for an inkjet printer [11], and the gamut was controlled by an alpha parameter which was interactively selected with a visualization system. Because it is difficult to determine the optimal parameter values, Morovic proposed the segment maxima gamut boundary description (SMGBD) algorithm to compute media and device gamut [4]. However, sometimes there are no boundary points falling into certain segments, it must be created by interpolation method, which pulls errors into the gamut calculations.

In general, the analytical algorithms can be used neither for all types of color devices, nor for the color images, while, for the empirical algorithms, the calculated boundary points are not very precise, because some inside-gamut boundary points may be mistaken for boundary points. In the paper, a new gamut description algorithm is proposed for calculating liquid crystal display (*LCD*) monitors'  3D and 2D gamut. Similar to Herzog's algorithm, the 3D* CIELAB* gamut is deemed as a deformed hexahedron, and the relationship between device and* CIELAB* gamut is analyzed and established, but it should be noted that the calculation process is far less complicated. For the experimented* LCD* monitor, the gamut boundary points are firstly found out in* RGB* space, and these obtained points are the true boundary points which are on the surface of* RGB* cube. And then the* RGB* boundary points are converted into* CIELAB* values, while the 3D or 2D gamuts are described based on these boundary points.

## 3. Three-Dimensional* CIELAB* Gamut Description

During the gamut description for* LCD* monitors, the device color of* RGB* signals are controlled by users to form the sample data, and the corresponding* CIELAB* values are obtained by measuring the screen when the* RGB* colors are displayed. In general, the calculation of monitor gamut in* CIELAB* space can be divided into three steps, which are* RGB* sample data generating,* CIELAB* data measuring, and 3D or 2D gamut calculation.

### 3.1. Generating the Sample Data

In order to obtain the monitor's* CIELAB* gamut boundary, the colors around the device gamut boundary should be selected to generate the sample data. Thus, for color monitors, the colorant gamut boundary is actually the outer surface of* RGB* cube, and the sample colors should be collected from it. With respect to the points on the top of* RGB* cube surfaces, there is at least one or two signals maintaining the maximal or minimal values.

If the *R*, *G*, and *B* values are ranging within 0~255 or normalized to 0~1, a certain amount of sample colors can be obtained by dividing the* RGB* cube surfaces' two colorants into *m* and *n* sections as below:

(1) Osample={u+v ∣ u,v∈[R,G,B],[u]={0,1m,2m,…,1},[v]={0,1n,2n,…,1}}.

When the* RGB* sample signals are sent to the displaying driver, they will be displayed on the monitor, and the spectral transmittance is measured by using a spectrophotometer. Then the* CIEXYZ* Tristimulus can be calculated with the following:

(2) X=K∫380780S(λ)x−(λ)τ(λ)dλ,Y=K∫380780S(λ)y−(λ)τ(λ)dλ,Z=K∫380780S(λ)z−(λ)τ(λ)dλ,K=100∫380780S(λ)y−(λ)R(λ)dλ,

where *S*(*λ*) is relative spectral power distribution of the illuminant, $\overset{-}{x}(\lambda )$, $\overset{-}{y}(\lambda )$, and  $\overset{-}{z}(\lambda )$ are the color-matching functions for* CIE* 2° standard observer (1931), and *τ*(*λ*) is the spectral transmittance of color patch, while the* CIELAB* values can be converted from the* CIEXYZ* values using the following:

(3) L∗=116f(YYn)−16,a∗=500[f(XXn)−f(YYn)],b∗=200[f(YYn)−f(ZZn)],

where *X*_*n*_,  *Y*_*n*_, and *Z*_*n*_ are the* CIEXYZ* tristimulus values of the reference white point, and the function *f* is defined as follows:

(4) $$\begin{array}{c} f\left(t\right)=\{\begin{array}{cc} t^{1/3} & \text{if}\;t>{\left(\frac{29}{6}\right)}^{3}\\ \frac{1}{3}{\left(\frac{29}{6}\right)}^{2}t+\frac{4}{29}\;\; & \text{otherwise}. \end{array}\;\; \end{array}$$

### 3.2. Calculation of 3D* CIELAB* Gamut

When a certain amount of sample colors are obtained, there will be many scattered points distributed in* RGB* or* CIELAB* space. The outer surface of* RGB* cube is the device gamut boundary with regular shape, and it will be severely deformed when converted into* CIELAB* space. For example, if the monitor's* RGB* cube is uniformly sampled by setting *m* = *n* = 9, in other words every colorant evenly changes in the range of [0 32 64 96 128 160 192 224 255], there will be 9 scattered points on each of the* RGB* cube edges.

**Figure 1:**
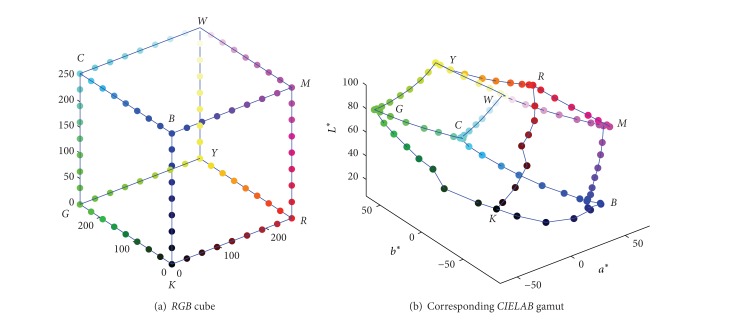
Gamut boundary formed by 12 edges of* RGB* cube.

The deformation between* RGB* cube and 3D* CIELAB* gamut can be analyzed by comparing the distribution of those 12 edges in these two color spaces.  Figure 1(a) is the device* RGB* gamut boundary formed by its 12 edges, and its corresponding* CIELAB* gamut boundary points is shown in Figure 1(b). It is obvious that the colorimetric* CIELAB* gamut looks like a deformed hexahedron in [7], whereas the deformation degree varies in different locations or for different devices.

**Figure 2:**
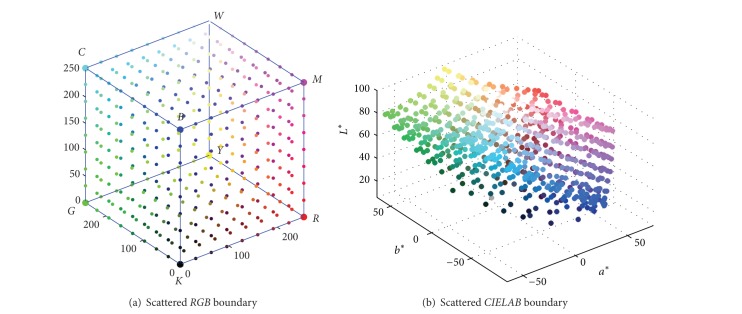
Scattered samples in colorant and colorimetric space.

To get the complete 3D gamut description, the six faces of the* RGB* cube should be converted into* CIELAB* space. Among the sampling points above, as only a part of them located on the outer surface of gamut, the* RGB* boundary points should be firstly extracted from the six* RGB* cube faces, and they are depicted in Figure 2. It can be seen that if all these 3D scattered points are smoothly connected, the complete and closed 3D gamut can be obtained.

With those obtained boundary points in* CIELAB* space,* Delaunay* triangulation technique can be used to link them, and the final completed 3D gamut boundary is composed of a quantity of triangular facets. Since the gamut surface is partitioned into planar triangles, the volume can be calculated when an interior point is assigned. For example, when the center point* O*  (50, 0, 0) in* CIELAB* space is selected, and all the vertices within each surface triangle are connected with the center point, then the* CIELAB* gamut is divided into many tetrahedrons. The overall gamut volume is the sum of the tetrahedrons' volumes, while each tetrahedron's volume can be calculated as below:

(5) $$\begin{array}{c} V=\frac{1}{6}\left\lfloor \begin{array}{cccc} 1 & 1 & 1 & 1\\ 50 & L_{1}^{*} & L_{2}^{*} & L_{3}^{*}\\ 0 & a_{1}^{*} & a_{2}^{*} & a_{3}^{*}\\ 0 & b_{1}^{*} & b_{2}^{*} & b_{3}^{*} \end{array}\right\rfloor , \end{array}$$

where *L*_1_*a_1_*b_1  _*, *L*_2_*a_2_*b_2_*, and *L*_3_*a_3_*b_3_* are three surface vertices of the tetrahedron.

## 4. Two-Dimensional Gamut Boundary for Gamut Mapping Algorithms

Because gamut mapping is commonly carried out in 2D gamut boundary, such as CIE-a*b* or CIE-C*L* gamut boundary, the 2D gamut boundary should be accurately determined. The 2D gamut is a cross- section of 3D* CIELAB* gamut; thus the 2D gamut boundaries can be determined by slicing the 3D gamut along constant-lightness plane or constant-hue-angle plane.

However, because the 3D boundary points do not distribute uniformly, when the scattered 2D boundary points are interpolated from the nearby 3D boundary points, there will be lots of errors caused. We propose a new method to calculate the 2D gamut boundary, in which the 2D boundary points are firstly found within the* RGB* cube, and then their* CIELAB* values are converted which are used to describe the gamut boundary in CIE-a*b* or CIE-C*L* coordinates.

### 4.1. *CIE*-a*b* Gamut Boundary Calculation

The* RGB* cube has eight vertices, twelve edges, and six faces. For color monitors, the lightness (*CIE*-L* values) of the vertices in the* RGB* cube varies greatly. Take the widely used ICC profile of* sRGB,* for example; the* RGB* cube's eight vertices'* CIELAB* values are listed in Table 1, and it can be seen that the vertices' lightness values are arranged in a sequence of below: *W* > *Y* > *C* > *G* > *M* > *R* > *B* > *K*. Actually most of the* RGB* monitors accord with this discipline.

In addition, as the lightness changes continuously on the faces of* RGB* cube, when a specific lightness value is given, a constant-lightness plane will intersect with some of the* RGB* faces, while the intersection line corresponds to the CIE-a*b* gamut boundary line. To calculate the constant-lightness gamut boundary, the scattered boundary points should be firstly found, and the process can be described in the following steps.

(1) Compare the given lightness *l* with twelve edges' lightness, and find out the boundary points on the edges equal to the given lightness value. For example, if *L*_*C*_ ≤ *l* ≤ *L*_*W*_, which means that the lightness value *l* is within the range of edge* CW*, then it can be concluded that an intersection point with lightness *l* must exist in the edge* CW*.

The intersection point can be found by comparing the edge's sample colors. If two closet sample colors are expressed as *x* and *y*, as their* RGB* and* CIELAB* values are known, the intersection point *p*'s *R*values can be interpolated as below (the same for *G* and *B* values):

(6) $$\begin{array}{c} R_{p}=R_{x}+\frac{L_{p}^{*}{-L}_{x}^{*}}{L_{y}^{*}{-L}_{x}^{*}}\left(R_{y}-R_{x}\right). \end{array}$$

**Figure 3:**
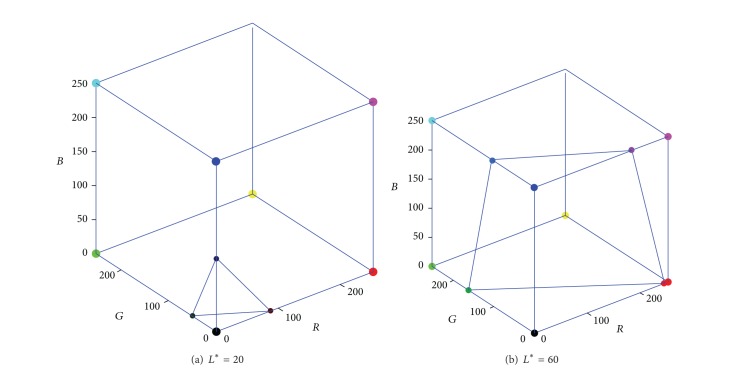
The scattered constant-lightness gamut boundary on* RGB* cube's edges.

**Figure 4:**
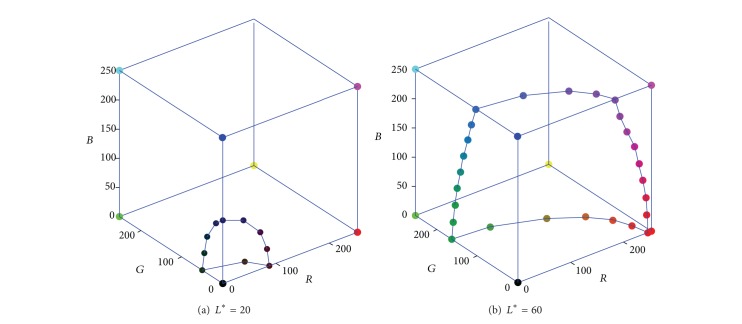
The scattered constant-lightness gamut boundary on* RGB* cube's faces.

**Figure 5:**
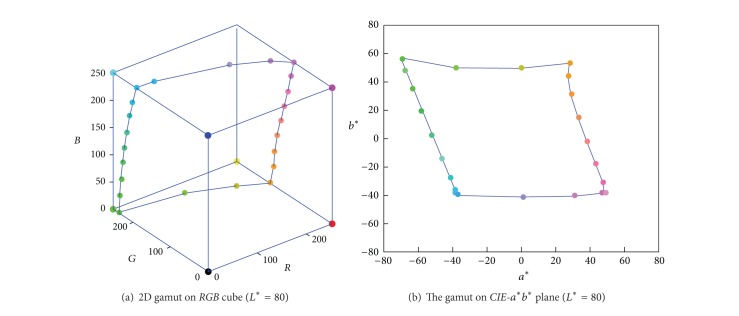
The constant-lightness gamut on* RGB* cube and* CIELAB* space.

(2) For the constant-lightness lines on the* RGB* cube surface, there are generally three or four edges which contain the intersection points among all the twelve edges. As shown in Figure 3, when the edge intersection points are connected, the constant-lightness lines may form a triangle (*CIE*-L* = 20) or quadrangle (*CIE*-L* = 60).

(3) Because the constant-lightness gamut boundary is a closed and smooth curve, several boundary points are calculated from the corresponding cube faces, for the purpose of obtaining the full description of the gamut boundary. The face intersection points can also be found by comparing the sample colors of the* RGB* cube face, which is similar to the process of calculating edge intersection points. Thus, when all the face boundary points are connected, the two constant-lightness gamut boundaries of Figure 3 are described with more details in Figure 4.

(4) After the scattered gamut boundary points in* RGB* space are calculated, the CIE-a*b* gamut boundary can be obtained by converting the boundary points from* RGB* space to* CIELAB *space. There are actually several color conversion models, such as 3D interpolation [12–14], polynomial regression [15, 16], and neural network [17, 18]. In the paper, the polynomial regression method is used because of the model's precision and the quantity of sample colors, and the utilized polynomials are expressed as below:

(7) P(R,G,B)=c0+c1R+c2G+c3B+c4RG+c5GB +c6RB+c7R2+c8G2+c9B2+c10RGB +c11R2G+c12R2B+c13G2R+c14G2B+c15B2R +c16B2G+c17R3+c18G3+c19B3,

where *c*_*i*_ (*i* = 0,1,…, 19) are the coefficients which can be determined by the least square method [19, 20]. Take one constant-lightness boundary, for example; it is depicted in* RGB* and* CIELAB *spaces respectively, as shown in Figure 5.

### 4.2. *CIE*-C*L* Gamut Boundary Calculation

The CIE-C*L*gamut boundary can be seen as a cross-section of the constant-hue-angle plane with 3D* CIELAB* gamut, and it is widely used in gamut mapping process. As most of the 3D gamut is calculated by the scattered sample data with interpolation method, it is hard to get the line boundary point directly using the continuous 3D gamut. Similar to the calculation of constant-lightness gamut, the constant-hue-angle boundary points are firstly calculated in* RGB* cube and then converted into* CIELAB* color space and described in the CIE-C*L* coordinate, where the CIE-C* is calculated from CIE-a* and CIE-b* values:

(8) $$\begin{array}{c} C^{*}=\sqrt{b^{*2}+b^{*2}}. \end{array}$$

During description of the 2D CIE-C*L* gamut, the scattered boundary points are firstly calculated and then connected with Bezier curves or straight lines. Although the boundary looks smoother when Bezier curves are used, the computing efficiency is inferior to the straight lines, which is very important for the gamut mapping process. Thus, the CIE-C*L* gamut boundary is connected using straight lines in this paper.

## 5. Experiments

In experiment, the gamut boundaries of a Toshiba* LCD* monitor are calculated. Firstly, the* RGB* device color space is uniformly divided, and the red, green, and blue signals all range within [0 32 64 96 128 160 192 224 255]; thus there are 9^3^ = 729 sample colors totally. And then the sample* RGB* colors are displayed on the* LCD* monitor; the corresponding* CIELAB* values are measured by using the spectrophotometer *X-Rite DTP*94. At last, the monitor's 3D* CIELAB* gamut and two other 2D gamut boundaries are described using the algorithm proposed in the paper.

**Figure 6:**
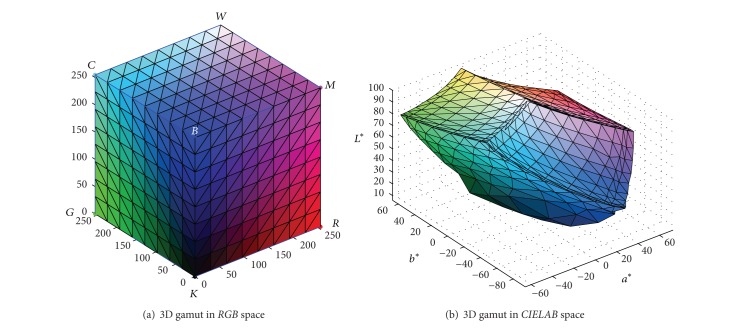
The continual 3D gamut in* CIELAB* of Toshiba monitor.

As shown in Figure 6, the 3D gamut is described in* CIELAB* color space, and it looks like a deformed hexahedron as Herzog proposed. Compared with the initial* RGB* cube, the* CIELAB* gamut also has eight vertices and six continual curved faces, although most of sample colors' relative positions have changed. From the described* CIELAB* gamut, the overall displaying capacity of the monitor can be evaluated, which will help to select the right gamut mapping algorithms.

Bakke et al. proposed a method for evaluating different gamut boundary calculating algorithms [21], and the two testing algorithms are compared by using a parameter of gamut volume mismatching rate, which is expressed in the following:

(9) $$\begin{array}{c} r_{i}=\frac{V\left(G_{i}/G_{\text{ref}}\right)+V\left(G_{\text{ref}}/G_{i}\right)}{V\left(G_{\text{ref}}\right)}, \end{array}$$

where *r*_*i*_ represents the gamut volume mismatching rate, *V*(*G*_ref_) is the volume of reference gamut, and *V*(*G*_*i*_/*G*_ref_) is the volume of colors which are inside of gamut *G*_*i*_ but outside of gamut *G*_ref_. In experiment, the gamuts determined by our algorithm are selected as reference gamut, and three famous gamut description algorithms, SMGBD, convex hull, and alpha shape, are employed as a contrast. Consequently, the mismatching rates are 3.2%, 2.4%, and 3.7%, respectively, which indicate that our algorithm's accuracy is very close to the three successful algorithms.

**Figure 7:**
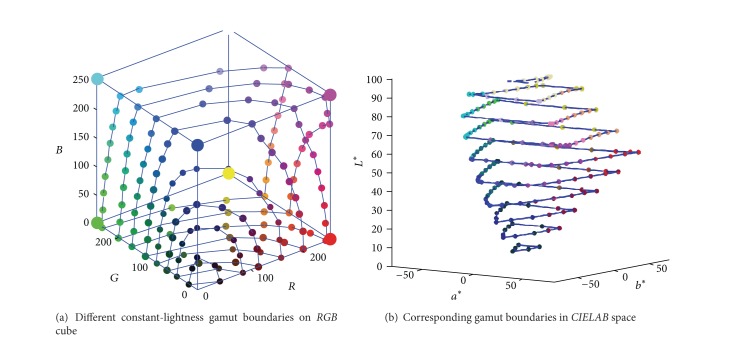
The 2D gamut boundary of different lightness.

**Figure 8:**
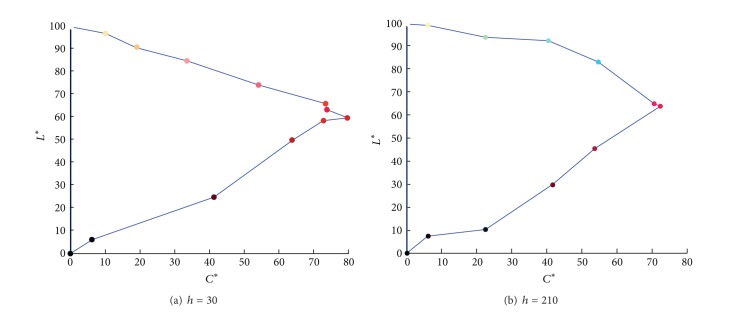
The 2D gamut with different hue angle.

Additionally, two kinds of 2D gamut mapping boundaries, CIE-a*b* and CIE-C*L* boundaries, are calculated using the method described in Sections 4.1 and 4.2. For the testing monitor, several constant-lightness lines with the lightness from 10 to 95 are found in* RGB* cube and then described in* CIELAB* space, as shown in Figure 7. Similarly, two CIE-C*L* boundaries are listed in Figure 8, and the hue angles are 30, 210, and 330 respectively.

## 6. Conclusions

The gamut of monitor is generally bigger than printers; thus when a displayed image is printed, gamut mapping should be performed in advance. Gamut mapping is the technique of replacing nonprintable colors by printable ones, conserving the appearance of an image. There are two major reasons which influence the gamut mapping precision, selection of gamut mapping algorithms and the calculation of gamut boundaries. Because many successful GMAs are developed for various images and color devices, it is very significant to improve the accuracy of gamut boundaries. In the paper, a new gamut boundary algorithm for color devices is proposed, and an* LCD* monitor is tested with having the 3D and 2D gamut boundaries described. In experiment, the described 3D* CIELAB* gamut boundary has regular-shaped outer surface, and the 2D CIE-a*b* and CIE-C*L* boundaries both have smooth boundary lines. For the purpose of precision evaluation, our algorithm is compared with other famous gamut description algorithms, and the result shows that the gamut volume difference is very little, which indicates that this algorithm is acceptable. Besides, it should be noted that, although the color monitor's gamut is described in experiment, the proposed gamut boundary calculating algorithm can also be applied to other color devices, such as cameras, scanners, or printers.

## Figures and Tables

**Table 1:** *RGB* and *CIELAB* values of the vertices for *sRGB.ICC. *

Color	*RGB *	L*	a*	b*
*K*	(0, 0, 0)	0	0	0
*B*	(0, 0, 255)	30	67	−128
*R*	(255, 0, 0)	55	79	65
*M*	(255, 0, 255)	60	91	−79
*G*	(0, 255, 0)	88	−81	71
*C*	(0, 255, 255)	91	−53	−34
*Y*	(255, 255, 0)	98	−18	83
*W*	(255, 255, 255)	100	−2	−19
